# Label-free quantitative proteomic analysis of insect larval and metamorphic molts

**DOI:** 10.1186/s12861-020-00227-z

**Published:** 2020-11-24

**Authors:** Weiye Si, Qingjie Wang, Yu Li, Dujuan Dong

**Affiliations:** 1 Shandong Provincial Key Laboratory of Animal Cells and Developmental Biology, School of Life Science, Shandong University, Qingdao, 266237 China; 2 Laboratory of Basic Medical Sciences, Qilu Hospital, Shandong University, Jinan, 250012 China

**Keywords:** Molting, Metamorphosis, Proteomics, Holometabolous insects

## Abstract

**Background:**

Molting is an essential biological process occurring characteristic times throughout the life cycle of holometabolous insects. However, it is not clear how insects determine the direction of molting to remain status quo or to initiate metamorphosis. To explore the functional factors that determine the direction of molts, liquid chromatography-mass spectrometry was used to identify the molecules involved in larval and metamorphic molting, and the differentially expressed proteins (DEPs) were compared in the two processes.

**Results:**

There were 321 and 1140 DEPs identified in larval and metamorphic molting process, respectively. Bioinformatics analyses show that the amino sugar pathway was up-regulated in both processes. The up-regulated protease contributed to the metamorphosis. In addition, several proteins with different expression patterns in larval-larval and larval-pupal transitions, including Endochitinase, GRIM-19 (Genes associated with retinoid-IFN-induced mortality-19), IDE (Insulin-degrading enzyme), Sorcin (Soluble resistance related calcium binding protein), OBP (Odorant-binding protein-2 precursor), TRAP1(Tumor necrosis factor receptor associated protein-1), etc., were further identified by parallel reaction monitoring, which may play diverse functions in larval-larval and larval-pupal transitions.

**Conclusions:**

These results provide a proteomic insight into molecules involved in larval and metamorphic molts, and will likely improve the current understanding of determination of direction of molts.

**Supplementary Information:**

The online version contains supplementary material available at 10.1186/s12861-020-00227-z.

## Background

Insects are a dominant terrestrial group on the Earth with wide distribution and tremendous amount. For the holometabolous class of insects, metamorphosis produced distinct morphology between larva and adult, which enhanced their adaptability to environment, and attributed to the evolutionary success [1]. Characteristic times molts segmented the lives of complete metamorphic insects into three major life stages, including larvae, pupae, and adults. Previous larval molts progress the larva from one instar to the next, during which a new cuticle secreted by the monolayer of epidermal cells and the old exoskeleton shed [2]. At the end of final larval instar, metamorphic molts switch the molting direction to pupa to adult. In addition to the replacement of old and new cuticle similar to the larval molts, degradation of larval tissues and remodeling of adult tissues occurred during the metamorphic molts [3–5]. How insects determine the direction of molts, to maintain the status quo or to enter metamorphosis, is one of the most frequently stated problems in research about insect’s physiology and development.

Existing researches on insect hormones have provided important information on the mechanism regulating molting direction. 20-hydroxyecdysone (20E) evokes the molts through signaling cascade, while the presence or absence of Juvenile hormone (JH) determines whether the larvae progress through their instars or formats pupa [6]. In the presence of JH, 20E directs larval molting. However, when larva acquired sufficient nutrients to survive metamorphosis and reached a “critical weight”, circulating JH was removed [7]. Then “pupal specifier”, such as broad, came up at the time of pupal commitment in response to 20E and opened up metamorphosis through inducing the transcription factors needed for pupal differentiation [8]. Meanwhile, the biosynthesis and secretion of ecdysone are coordinately regulated by plethora of signaling pathways, including prothoracicotropic hormone, insulin/insulin-like growth factors, neuropeptides, JH and 20E, transforming growth factor β, and hedgehog, etc. [9]. These researches have provided a schematic of how hormones regulate the determination of nature of molts. However, not all of the details have been resolved. What other molecules involve in the process of molting and metamorphosis still needs further identification.

A major technological driver in the identification has been the development of high-sensitivity protein mass spectrometry by allowing made the absolute quantification of proteins. Several proteomic studies have led to identify molting related proteins. For example, proteomic analysis was used to investigate the proteins in molting fluids before pupation and eclosion in the silkworm, *Bombyx mori* [10]. Proteins in the prothoracic glands (PGs) over the final larval stage have also been characterized in *B. mori* to identify the molecular nature and variety of receptors that through the ecdysteroidogenesis [11]. However, large scale investigation on changes in protein abundance of whole body during status quo and metamorphic molts hasn’t been reported.

Here, we used *Helicoverpa armigera*, a holometabolous crop pest that belongs to Lepidoptera, the species with the greatest impacts on agriculture, as a model to explore more molecules involved in larval-larval and larval-pupal transitions.

The proteome of fifth and sixth instar larvae at feeding and molting stages were identified by liquid chromatography-mass spectrometry (LC-MS/MS) respectively. Bioinformatics analysis was used to compare the DEPs which may be involved in larval molting and metamorphic molting. The results show that the up-regulated proteins were enriched in the amino sugar metabolism pathway both in larval molting and metamorphic molting, which may be related to the metabolism of chitin involved in the replacement of integument. In addition, the up-regulated proteins in the larval molting are also enriched in the lipid transport process, while the up-regulated proteins in the metamorphic molting stage mainly were associated with protein catabolism. Further, several types DEPs with different expression patterns in larva molting and metamorphosis molting were analyzed by parallel reaction monitoring (PRM) to validate the results of the proteomics. By conducting the research, we may refine the mechanism of determination of direction of molts and find the novel molecular targets to control the pest effectively.

## Results

### Larva molting and metamorphic molting in cotton worm

**Fig. 1:**
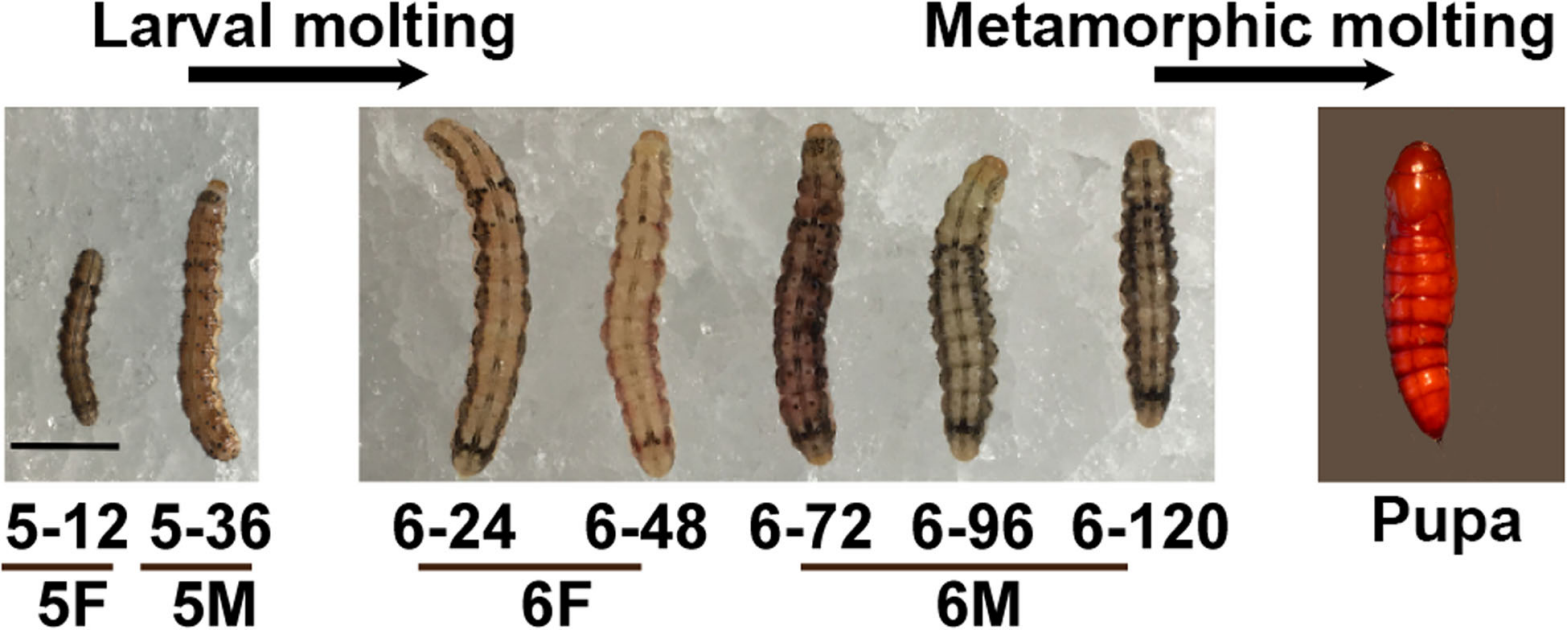
Representative images of cotton worm (*H. armigera*) during feeding and molting stages. 5–12, 5–36: 12, 36 h after ecdysis into 5th instar; 6–24, 6–48, 6–72, 6–96, 6–120: 24, 48, 72, 96, 120 h after ecdysis into 6th instar. 5F, fifth instar feeding stage; 5 M, fifth instar molting stage; 6F, sixth instar feeding stage; 6 M, sixth metamorphic molting stage. Scale bar = 1 cm

Larva molts segment the life history of cotton worm into six instars, and then the metamorphic molting allows the sixth instar larva transform into the pupae stage (Fig. 1). To identify proteins related to larval molting and metamorphic molting, the feeding and molting stages of fifth and sixth instar larva were sampled for protein preparation and quantitative proteomics analyses respectively.

### Identification of differentially expressed proteins

**Fig. 2:**
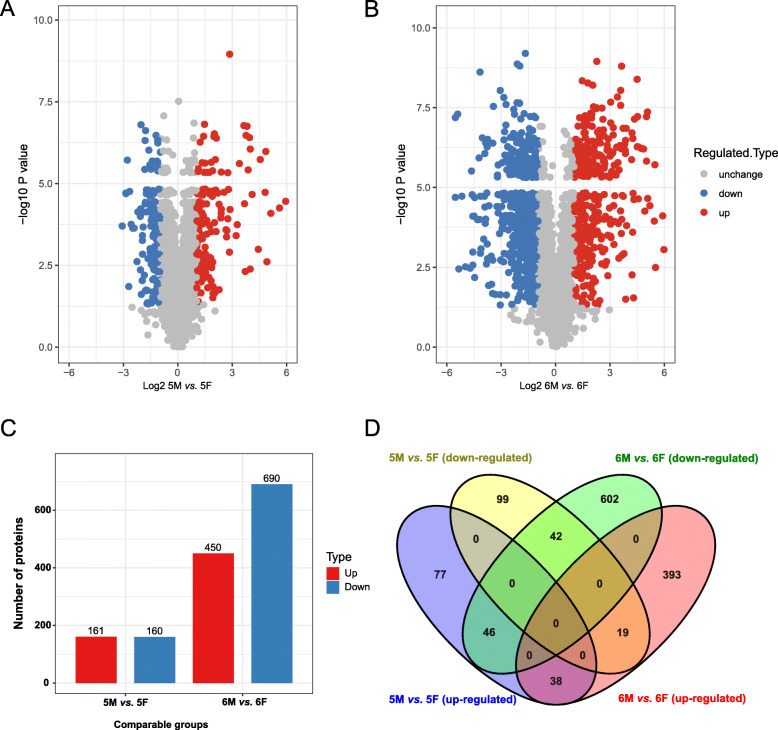
Distribution of DEPs of *H. armigera* in stages of 5F, 5 M, 6F and 6 M. Volcano plots indicating DEPs from 5 M *vs*. 5F group (**a**) and 6 M *vs*. 6F group (**b**). The horizontal coordinate represented the fold change (logarithmic transformation at the base of 2), and the vertical coordinate represented the significant difference *p* value (logarithmic transformation at the base of 10). Red dots mean the up-regulated proteins, blue dots mean the down-regulated proteins and gray dots mean the unchanged proteins; **c** The numbers of up-regulated (red) and down-regulated (blue) DEPs from two comparable groups; **d** Venn diagram displayed the DEPs of *H. armigera* in stages of 5F, 5 M, 6F and 6 M

DEPs occur during different growth and development stages of *H. armigera*. Using mass spectrometry technology, 3386 quantifiable proteins were obtained from the samples. In fifth instar molting stage (5 M) group compared with fifth instar feeding stage (5F) group, there were 321DEPs, of which 161 were up-regulated and 160 were down-regulated (Fig. 2a and c, FC ≥ 2 or FC ≤ 0.5, *P* < 0.05). Compared with sixth instar feeding stage (6F) group, there were 450 up-regulated and 690 down-regulated DEPs in sixth metamorphic molting period (6 M) group (Fig. 2b and c, FC ≥ 2 or FC ≤ 0.5, *P* < 0.05). The numbers of 77, 99, 393 and 602 unique proteins were found in the groups of 5 M versus (*vs*.) 5F (up-regulated), 5 M *vs*. 5F (down-regulated), 6 M *vs*. 6F (up-regulated) and 6 M *vs*. 6F (down-regulated), respectively (Fig. 2d). A total of 38, 42, 46, and 19 same proteins were identified in two groups, including the groups of 5 M *vs*. 5F (up-regulated) and 6 M *vs*. 6F (up-regulated), the groups of 5 M *vs*. 5F (down-regulated) and 6 M *vs*. 6F (down-regulated), the groups of 5 M *vs*. 5F (up-regulated) and 6 M *vs*. 6F (down-regulated), and the groups of 5 M *vs*. 5F (down-regulated) and 6 M *vs*. 6F (up-regulated), respectively. However, there were no common elements among all the four groups (Fig. 2d). The detailed information of all identified proteins is shown in Additional file 1.

### Gene ontology (GO) enrichment analysis

**Fig. 3:**
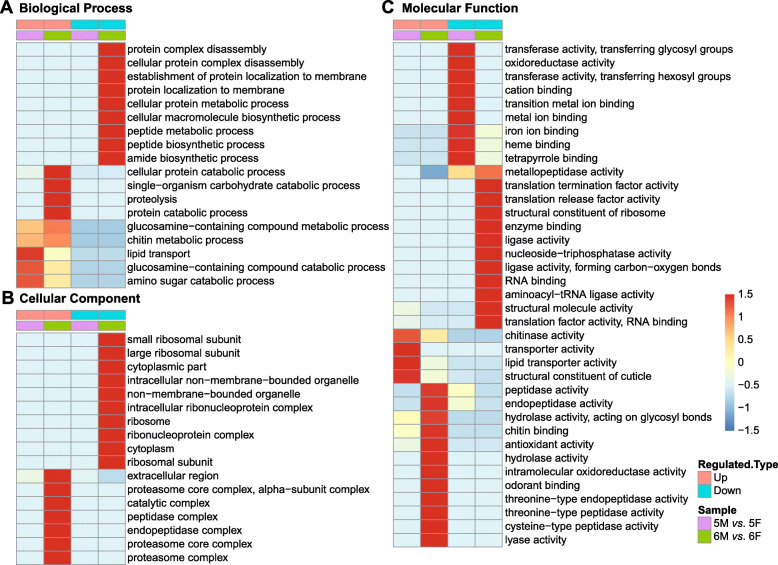
Cluster analysis heat map based on GO enrichment from 5 M *vs*. 5F group and 6 M *vs*. 6F group. **a** Biological process; **b** Cellular component; **c** Molecular function

To further identify their functions, different categories were performed to annotate all the DEPs. GO is an important bioinformatics analysis tool, which were classified into three categories: biological process (BP), cellular component (CC) and molecular function (MF). After GO enrichment analysis of DEPs in different comparison groups, the correlation of them was compared using the clustering analysis and the results were presented using a heat map (Fig. 3). The vertical direction is the description of DEPs enrichment related functions, and the horizontal direction indicates the results of enrichment test of the different groups. Different color blocks indicate the degree of enrichment, and the deeper the red color is, the higher the enrichment degree is. There were significant differences in GO terms level 2 among the four groups.

In BP categories: the up-regulated DEPs from 5 M *vs*. 5F group were associated with terms including lipid transport, glucosamine-containing compound catabolic process, and amino sugar catabolic process. The up-regulated DEPs from 6 M *vs*. 6F group were mainly clustered into cellular protein catabolic process, single-organism carbohydrate catabolic process, proteolysis and protein catabolic process. The remarkable thing is that for up-regulated DEPs, “glucosamine-containing compound metabolic process” and “chitin metabolic process” showed a certain degree of enrichment in both 5 M *vs*. 5F and 6 M *vs*. 6F group (Fig. 3a).

In CC categories: the up-regulated DEPs from 6 M *vs*. 6F group displayed obvious enrichment in extracellular region, proteasome core complex, and alpha-subunit complex. Meanwhile, the down-regulated DEPs from 6 M *vs*. 6F group were clustered into small ribosomal subunit and large ribosomal subunit (Fig. 3b).

In MF categories: the up-regulated DEPs from 5 M *vs*. 5F group were mainly clustered into chitinase activity, transporter activity and structural constituent of cuticle. The up-regulated DEPs from 6 M *vs*. 6F group were mainly related to some enzyme activities, such as peptidase, endopeptidase, hydrolase, etc. Meanwhile, the down-regulated DEPs from 5 M *vs*. 5F group mainly participated in transferase activity, transferring glycosyl groups and oxidoreductase activity, etc. The down-regulated DEPs from 6 M *vs*. 6F group were linked to the translation related activity, such as translation termination factor activity and translation release factor activity, etc. The common functional annotations were found in both larval molting group and metamorphic molting group. The up-regulated DEPs displayed obvious enrichment in chitinase activity, and the down-regulated DEPs were clustered into metallopeptidase activity (Fig. 3c).

These results implied that the glucosamine-containing compound metabolic and chitin metabolic may be associated with molting process and the DEPs between larval molting and metamorphosis molting may be the key factors to determine the molting properties.

### KEGG (Kyoto encyclopedia of genes and genomes) enrichment analysis

In order to further understand the pathway information associated with the molting process of the cotton bollworm, KEGG enrichment analysis was performed on the DEPs in each comparison group.

**Fig. 4:**
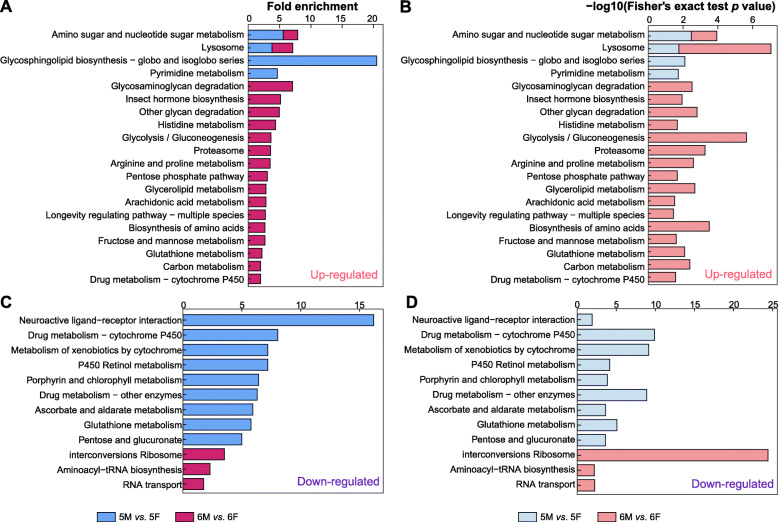
KEGG enrichment analyses of DEPs from two comparison groups of 5 M *vs*. 5F and 6 M *vs*. 6F in *H. armigera*. **a** represented the enrichment scores of the up-regulated pathway; **b** was obtained using a negative logarithm (−log10 Fisher’s exact test p value) of up-regulated pathway; **c** represented the enrichment scores of down-regulated pathway; **d** was obtained using a negative logarithm (−log10 Fisher’s exact test p value)

Up-regulated amino sugar and nucleotide sugar metabolism and lysosome pathway was contributed in both 5 M *vs*. 5F and 6 M *vs*. 6F groups (Fig. 4a), while there were no common down-regulated pathways in the two groups (Fig. 4c). Importantly, the two groups also contained specific pathways, respectively. Cluster analysis of up-regulated pathways showed that the proteins from “Glycosphingolipid biosynthesis” and “Pyrimidine metabolism” were enriched in 5 M *vs*. 5F group, the proteins from “Glycosaminoglycan degradation” and “Insect hormone biosynthesis” were enriched in 6 M *vs*. 6F group. In the down-regulated pathway, “Neuroactive ligand-receptor interaction” and “Ribosome” were enriched significantly in 5 M *vs*. 5F group and 6 M *vs*. 6F group respectively. The results showed that there were significant differences in larval molting and metamorphic molting process.

### PRM validation analysis

**Fig. 5:**
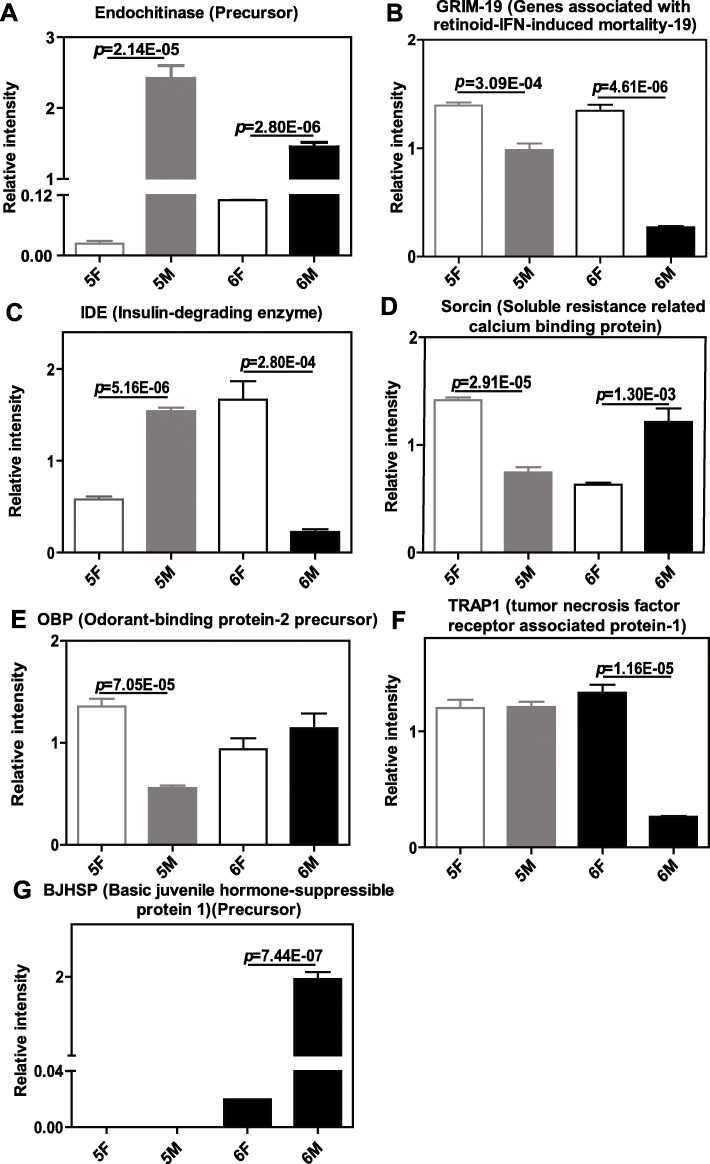
Using PRM, seven potential DEPs were validated from two comparison groups of 5 M *vs*. 5F and 6 M *vs*. 6F in *H. armigera*. They showed significant difference between different groups (*P* < 0.05)

To confirm the authenticity and accuracy of the proteomic analysis, seven candidates DEPs in larval and metamorphic molts, which have been proved to play important roles in various life processes, were quantified by PRM assay. The results based on PRM supported the shotgun proteomics discovery and revealed that Endochitinase (Precursor), GRIM-19 (Genes associated with retinoid-IFN-induced mortality-19), IDE (Insulin-degrading enzyme), Sorcin (Soluble resistance related calcium binding protein), OBP (Odorant-binding protein-2 precursor), TRAP1(Tumor necrosis factor receptor associated protein-1), and BJHSP (Basic juvenile hormone-suppressible protein 1)(Precursor) were altered expression in different groups (Fig. 5). With reference from the figures, Endochitinase (Precursor) was significantly elevated in both 5 M *vs*. 5F and 6 M *vs*. 6F groups (*P* < 0.05, respectively). GRIM-19 was significantly decreased in both 5 M *vs*. 5F and 6 M vs. 6F groups (*P* < 0.05, respectively). IDE was obviously increased in 5 M *vs*. 5F group and decreased in 6 M *vs*. 6F group, while Sorcin had the opposite change trend (*P* < 0.05, respectively). OBP was only down-regulated in 5 M *vs*. 5F group, while the expression of TRAP1 was only down-regulated in 6 M *vs*. 6F group (*P* < 0.05, respectively). The altered expression of BHJSP was only up-regulated in 6 M *vs*. 6F groups (*P* < 0.05).

## Discussion

Using LC-MS/MS and bioinformatics analysis, the specific proteins of fifth and sixth instar larvae at feeding and molting stages were identified in the present study, which provided potential targets to the determination of molting properties and biological control of pest.

### Up-regulated amino sugar pathway was contributed in both larval molting and metamorphic molting

**Fig. 6:**
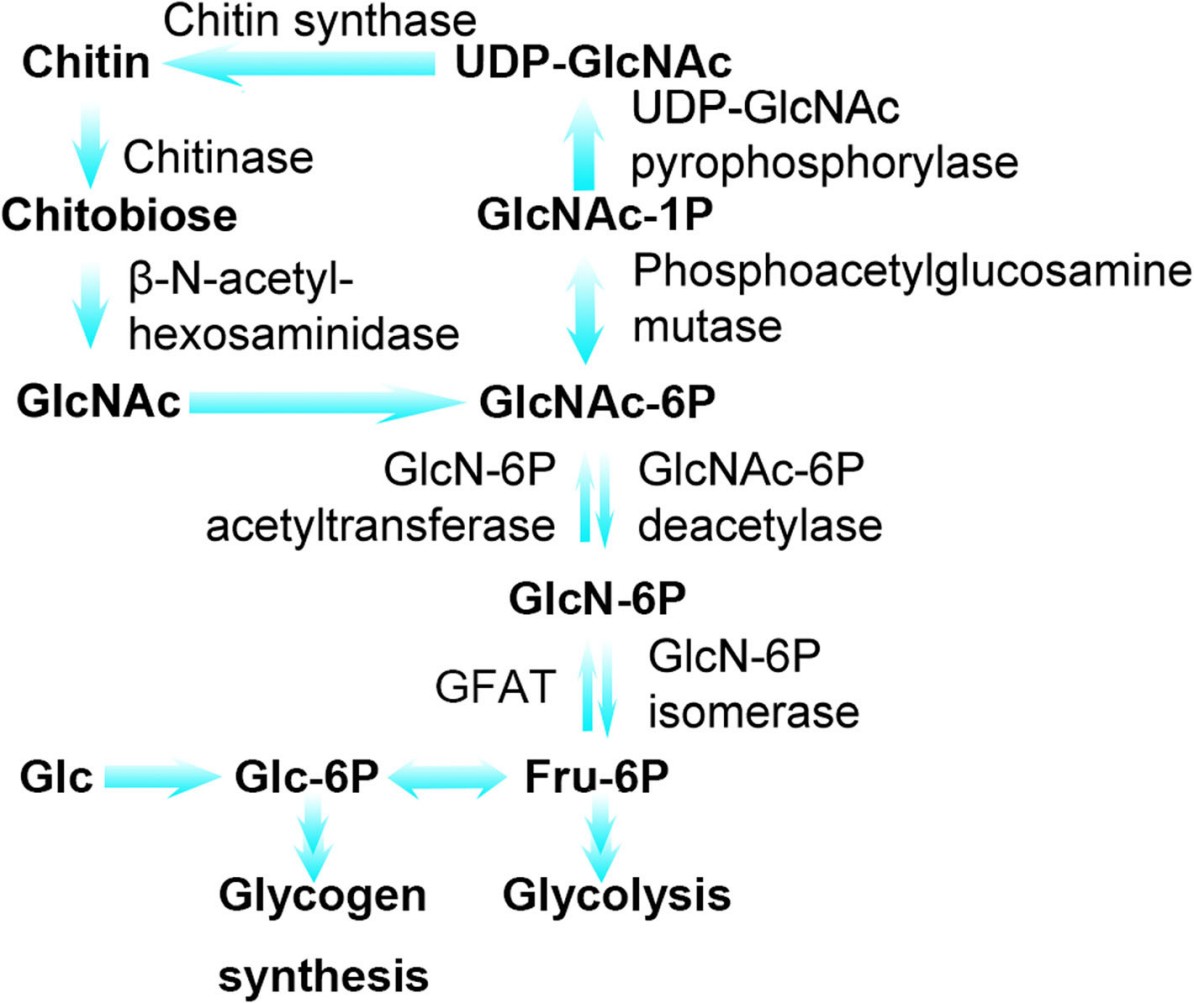
Amino sugar and nucleotide sugar metabolism pathway. The diagram was plotted based on the KEGG pathway database. Glc, Glucose; P, Phosphate; Fru, Fructose; GlcN, Glucosamine; GlcNAc, N-acetylglucosamine

GO analysis showed that the up-regulated proteins in larval and metamorphosis molting were enriched in the process of “glucosamine-containing compound metabolic” and “chitin metabolic” (Fig. 3a). In addition, KEGG analysis showed that the up-regulated proteins in the two groups were highly enriched in the amino sugar and nucleotide sugar metabolism pathway (Fig. 4a). As shown in KEGG PATHWAY DATABASE (https://www.genome.jp/kegg-bin/show_pathway?map=map00520&show_description=show), all above the processes were interrelated (Fig. 6). These DEPs mainly include chitinase, β-N-acetyl-hexosaminidase, phosphoacetylglucosamine mutase, N-acetylglucosamine-6-phosphate deacetylase (GlcNAc-6P deacetylase), etc.

On the one hand, these enzymes are involved in chitin metabolism. Chitin is a kind of N-acetyl-β-D-glucosamine (GlcNAc) linear polymer linked by β-1, 4-glycoside bond, which is the main component of cuticle and midgut membrane of insects. Its synthesis and degradation are very important for the growth and development of insects [12]. Previous studies in *Manduca sexta* have showed that the synthesis and activation of chitinolytic enzymes, including chitinase and β-N-acetyl-hexosaminidase, were increased by ecdysteroid [13]. The *M. sexta* chitinase gene was expressed only before larval-larval, larval-pupal and pupal-adult molting [14]. The LC-MS/MS and PRM analysis of this study showed that Endochitinase (Precursor) was significantly elevated in both 5 M *vs*. 5F and 6 M *vs*. 6F groups, which corroborated the findings of a great deal of the previous work in *M. Sexta*. Therefore, it seems that both larva molting and metamorphosis molting are involved in chitin metabolism, although they have different directions after molting.

On the other hand, UDP-GlcNAc, a product of hexosamine metabolism, can provide covalent modifying groups for O-GlcNAc modification. Chitinase and GlcNAc-6P deacetylase catalyze the metabolism of hexosamine to the direction of glycolysis, which will reduce UDP-GlcNAc in cells. This suggests that the decrease of UDP-GlcNAc may be related to the initiation of molting.

### Activity of protease is related to metamorphic molting

In the current study, compared with 5 M *vs*. 5F group, the up-regulated DEPs from 6 M *vs*. 6F group, including lysosomal aspartic protease, serine protease, and cysteine proteinase, were mainly clustered into cellular protein catabolic process, single-organism carbohydrate catabolic process, proteolysis and protein catabolic process in BP (Fig. 3a) and peptidase and endopeptidase activity in MF (Fig. 3c). A possible explanation for this might be that the hydrolytic activity of protease is necessary for larval tissue programmed cell death during metamorphosis. In accordance with the present results, prior studies in *B. mori* have shown that the high expression of cysteine-type cathepsins, aspartic-type cathepsins and metzincins occurs during metamorphic molting. In addition, they participate in the destruction of larval fat body during larval-pupal transition, and they are transcriptionally up-regulated by 20E signals [15]. Data from *H. armigera* have also noted that the cysteine-type cathepsin L participates in midgut apoptosis via caspase-1 [16].

Proteases are widely distributed in organisms. Besides protein hydrolysis, they also have other important physiological functions. Mutations for Carboxypeptidase D in *Drosophila* led to altered wing shape [17] and mating behavior in light [18]. In *H. armigera*, lysosomal aspartic protease cathepsin D promotes midgut apoptosis via the mature form inside cell, and promotes cell proliferation and reassociation of the adult fat body via secreting a pro-enzyme outside the cells as a ligand [19].

One of the issues that emerge from these findings is that up-regulated protease contributed to the initiation of metamorphosis.

### The DEPs in larva-larva and larva-pupa transit may be involved in the determination of molting properties

In the PRM analysis, we selected seven targets with different trends in the process of larva molting and metamorphosis molting. These targets may help us to find more proteins related to the determination of molting.

Endochitinase (precursor) was significantly elevated in both 5 M *vs*. 5F and 6 M *vs*. 6F groups (Fig. 5a). As we discussed above, this result corroborates a great deal of findings in *M. sexta* and *H. armigera*. The *M. sexta* chitinase is abundant during larval-larval, larval-pupal and pupal-adult molting [14]. The *H. armigera* chitinase presents in molting stages but is absent in the intermoult periods [20]. These findings may be due to the fact that chitin is the main component of cuticle, and chitinase is involved in the replacement of old and new epidermis. So molecules up-regulated in both larval-larval and larval-pupal processes in the present study may be involved in the “molting process” itself.

GRIM-19 is a subunit of mitochondrial respiratory complex I. Our previous study showed that GRIM-19 was highly expressed during the larval feeding stage, and the knockdown of GRIM-19 by RNA interference could induced programmed cell death, which imply that GRIM-19 plays roles in keeping the normal cellular growth [21]. Proteomics analysis results that the abundance of GRIM-19 decreased in both 5 M *vs*. 5F and 6 M *vs*. 6F groups are consistent with our previous observations, which suggested that the proteins enriched in feeding period may keep the normal cellular growth, and inhibit the molting process.

IDE is a zinc metalloprotease that can cleave insulin into inactive fragments. Knocking down IDE in Drosophila can decrease circulating sugar and reduce lifespan, which suggested a role for IDE in determining the level of insulin-like peptides that systemically activate insulin signaling [22]. 20E and insulin signals have been demonstrated to cross talk with each other to regulate insect development [23]. Insulin signaling pathway promotes larval growth and the ecdysteroid production in PGs [24]. 20E, in turn, counteracts insulin pathway to initiate apoptosis and metamorphosis [25]. The antagonism between hormones may be regulated by their titers at different development stages [26]. Our result showed that the expression of IDE was up-regulated in larval molting stage and down regulated in metamorphosis molting stage, which may change the activity of 20E by changing the concentration of insulin, so as to participate in molting process.

Sorcin is a small soluble penta-EF-family of calcium binding protein [27], which is associated with calcium (Ca^2+^) homeostasis by regulating Ca^2+^ channels [28, 29] or binding with Ca^2+^ directly [30]. 20E can trigger rapid fluctuation of Ca^2+^ through membrane receptors and regulate gene expression via a nongenomic signaling pathway [31]. Our result has showed that Sorcin was decreased in 5 M *vs*. 5F group and increased in 6 M *vs*. 6F group, which suggested that Sorcin may play crucial roles in the 20E regulated initiation of metamorphosis through changing Ca^2+^ level. In addition, sorcin can induce different signaling pathways by modulating the levels of important cellular proteins such as Akt [32], ERK1/2 (Extracellular regulated protein kinases) [33], MMPs (Matrix metalloproteinases) [34], caspases [35], which suggested that Sorcin was associated with the hormonal cross talk in insect development.

OBP, a family of small water soluble proteins containing around 120 to 150 amino acids, plays important role in locating food and mates [36]. The family proteins are able to bind and transport various hydrophobic odorant molecules to the odorant receptors, and then induce the olfactory signal transduction system [37]. In brown planthopper, OBP can be involved in nymph olfaction on rice seedlings, and have non-olfactory functions. Silencing OBP encoding gene resulted in strikingly high nymph mortality, which may be due to disruption of JH binding and transporting possibly carried out by OBP [38]. Expression patterns analysis showed that OBP has a nymph biased expression and then decreased in the final nymph stage before molting to adults. The present study shows a similar trend. The expression of OBP in feeding stage was higher than that in molting stage, and the altered expression of Odorant-binding protein-2 precursor was only found in 5 M *vs*. 5F group. Our results suggested that OBP may be associated with molts through their binding ability with JH besides its olfactory functions.

TRAP1 was identified as a novel protein that binds to the receptor for tumor necrosis factor through the yeast-based two hybrid, which shows strong homology to members of the 90-kDa family of heat shock proteins (Hsp90) [39]. Hsp90 are conserved chaperone proteins that protect the structure and function of proteins and play a significant task in cellular homeostasis and signal transduction [40]. In *H. armigera*, Hsp90 contribute to insect development by altering phosphorylation and protein interactions in the cross talk between 20E and JH [41]. The result of current study showed that the expression pattern of TRAP1 was in accordance with that of Hsp90 in the fat body of *H. armigera*, which suggests that TRAP1 may have similar function with Hsp90.

BJHSP is a storage protein identified in *Trichoplusia ni*, which can provide the biosynthetic precursors and energy for insect metamorphosis [42]. The mRNA abundance of BJHSP increased dramatically during the metamorphic larval stadium. Maintenance of a high JH titer caused a strongly suppression for the transcripts of BJHSP [43]. Our proteomic data showed that the expression of BJHSP was not detected in the fifth instar larvae, but only up regulated in the metamorphosis stage of the final instar larvae, which was consistent with the results in *T. Ni*. These results suggest that after JH is removed in the metamorphic larval stadium, its inhibition on BJHSP is released and BJHSP is up-regulated, which may be involved in the initiation of metamorphosis.

## Conclusions

In current study, we identified the functional molecules related to larval molting and metamorphosis molting at proteomic levels, and compared the DEPs in the two processes. The up-regulated amino sugar pathway was detected in both larval molting and metamorphic molting, which indicated that chitin metabolism and fluctuation of O-GlcNAc may be involved in the regulation of molts. The result has also shown that up-regulated protease contributed to the initiation of metamorphosis. In addition, several DEPs with different expression patterns in larva molting and metamorphosis molting, including Endochitinase (precursor), GRIM-19, IDE, Sorcin, OBP, TRAP1, and BJHSP were applied quantitative assays based on PRM. The results supported the shotgun proteomics, and indicated that they may play diverse functions in different mode of molts. Considering the possibility that DEPs may be detected due to normal development of insect, further work needs to be done to establish whether and how the DEPs are relevant to the determination of molting properties.

## Methods

### Insects

The cotton bollworms, *H. armigera*, were bought from Jiyuan Baiyun Industrial Co., Ltd., Henan, China, and were raised in the insectarium at 27 °C with a 14:10 h light-dark cycle. The Larvae were fed on the artificial diet described in previous work [44].

Fifth instar larvae eat for more than 30 h, and the head capsule slippage (HCS) occurred at about 5th 36 h. Then larvae shed the old cuticle and enter 6th instar, the final larval instar. After eating for more than 48 h, 6th larvae begin wandering at 72 h, then the legs contract and pupation at about 140 h. Considering 20E titer pulse [45] and according the phenotype, we prepared the samples at 5th instar feeding (5F, 12–24 h after ecdysis into 5th instar), 5th molting (5 M, 36 h after ecdysis into 5th instar), 6th instar feeding (6F, 12–60 h after ecdysis into 6 h instar), and 6th instar metamorphosis (72–120 h after ecdysis into 6 h instar) stages.

### Protein extraction and trypsin digestion

Firstly, the whole bodies of bleeding larvae at 5F, 5 M, 6F, 6 M stages were grinded in liquid nitrogen. Then the cell powder was sonicated three times on ice in the lysate buffer containing 8 M urea and 1% Protease Inhibitor Cocktail. After centrifugation (12, 000 g for 10 min at 4 °C), the supematant was harvested, and the Sigma Bicinchoninic Acid kit was used to measure the remaining protein concentration following the manufacturer’s instructions.

After reduction (5 mM dithiothreitol for 30 min at 56 °C) and alkylation (11 mM iodoacetamide in the dark at room temperature for 15 min), the protein solution was diluted with 100 mM tetraethylammonium bromide to urea concentration less than 2 M. Then the samples were trypsin-digested two times (1:50 trypsin-to-protein overnight and 1:100 for another 4 h) and fractionated by high pH reverse-phase HPLC using a Thermo Betasil C18 column (5 μm particles, 10 mm ID, 250 mm length).

### LC-MS/MS analysis

The enriched peptides were loaded onto a 15 cm length, 75 μm i.d. home-made reversed-phase analytical column after being dissolved in solvent A (0.1% formic acid). The gradient of solvent B (0.1% formic acid in 98% acetonitrile) increased from 6 to 23% in 26 min, from 23 to 35% in 8 min, and then climbing to 80% in 3 min and was held at 80% for 3 min. All operations were done on an EASY-nLC 1000 UPLC system at a constant flow rate of 400 nL/min.

After subjected to NSI source, the peptides were analyzed by tandem mass spectrometry (MS/MS) in Q Exactive™ Plus (Thermo) coupled online to the UPLC with 2.0 kV electrospray voltage. For full scan, the m/z scan range from 350 to 1800 was used. Intact peptides were detected in the Orbitrap at a resolution of 70,000 and then selected for MS/MS using NCE setting as 28. The ion fragments were detected in the Orbitrap at a resolution of 17,500. The data-dependent procedure was set as alternation between one MS scan followed by 20 MS/MS scans with 15.0 s dynamic exclusion. 5E4 was set for automatic gain control and 100 m/z was set for fixed first mass.

The mass spectrometry proteomics data have been deposited to the publicly accessible database PRIDE Archive (https://www.ebi.ac.uk/pride/) and can be accessed with the dataset identifier PXD020414.

### Database search

The resulting tandem mass spectra data were queried against our unpublished transcriptome data of *H. armigera* using the Maxquant search engine (v.1.5.2.8) concatenated with the reverse decoy database. Trypsin/P was specified as cleavage enzyme and up to 4 missing cleavages were allowed. The mass tolerance of 20 ppm was used for precursor ions in first search and 5 ppm in main search, and the mass tolerance of 0.02 Da was set for fragment ions. The fixed modification was carbamidomethyl on Cys and variable modifications contained acetylation modification and oxidation on Met. FDR was adjusted to < 1% and minimum score for modified peptides was set > 40.

UniProt-GOA database (http://www.ebi.ac.uk/GOA/) and InterProScan software (http://www.ebi.ac.uk/interpro/, version 5.14–53.0) was used to perform GO annotations. KAAS (http://www.genome.jp/kaas-bin/kaas_main, version 2.0) and KEGG Mapper (http://www.kegg.jp/kegg/mapper.html, version 2.5) were used to perform KEGG analysis. R Package pheatmap (https://cran.r-project.org/web/packages/cluster/, version 2.0.3) was used to generate the heat map of protein abundance.

### Functional enrichment

#### GO enrichment analysis

According to GO annotation, proteins were classified into three groups based on BP, CC and MF. For each category, the enrichment of differentially abundant proteins relative to all identified proteins was detected using a double-tailed Fisher’s precision test. GO with a revised *P* value of < 0.05 is considered significant.

#### Pathway enrichment analysis

Based on KEGG database, the enriched pathways to test the enrichment of the differentially expressed protein against all identified proteins were performed using a two-tailed Fisher’s exact test. The pathway was considered significant with a corrected *p*-value < 0.05. These pathways were classified into hierarchical categories through the KEGG website.

#### Enrichment-based clustering

Further hierarchical clustering were performed based on GO and KEGG. All the categories obtained after enrichment along with their *P* values were first collated, and followed filtering for those categories which were at least enriched in one of the clusters (*P* value < 0.05). The function x = −log10 (*P* value) was used to transform the filtered P value matrix and these x values were z-transformed for each functional category. One-way hierarchical clustering (Euclidean distance, average linkage clustering) in Genesis were used to cluster these z scores. The “heatmap.2” function from the “gplots” R-package was applied to visualize the cluster membership via a heat map.

### Parallel reaction monitoring (PRM)

The tryptic peptides were dissolved in 0.1% solvent A and injected into an EASY-nLC 1000 UPLC system, using a reversed-phase analytical column (15 cm length and 75 μm i.d.). The elution gradient was set as 6–23% solvent B for 38 min, 23–35% for 14 min, climbing to 80% in 4 min, and finally keeping at 80% for 4 min.

After subjected to NSI source, the peptides were analyzed by MS/MS in Q Exactive™ Plus coupled online to the UPLC with 2.0 kV electrospray voltage. A full mass spectrum at 35,000 resolution (AGC target 3E6, 20 ms maximum injection time, m/z 350–1000) was followed by up to 20 PRM scans at 17,500 resolution (AGC target 1E5, auto maximum injection time). PRM data were manually curated within Skyline (v.3.6).

### Statistical analysis

All experiments were performed in triplicate. Column charts and their statistical analysis were made using graph Pad Prism 5.0 software. Statistical comparisons between groups were estimated by Student’s t-test and *P* < 0.05 was considered statistically significant.

## Supplementary Information

**Additional file 1.** The detailed information of all identified DEPs in larval and metamorphic molts. The detail information of DEPs in two groups (5 M *vs*. 5F; 6 M *vs*. 6F), including protein accession, protein description, gene name, peptide number, matching scores, and functional enrichment analysis results.

## Data Availability

The datasets generated or analysed during this study are included in this published article and its supplementary information files. The raw mass spectrometry proteomics data have been deposited to the publicly accessible database PRIDE Archive (https://www.ebi.ac.uk/pride/) with the dataset identifier PXD020414.

## Abbreviations

20E: 20-hydroxyecdyson
DEPs: Differentially expressed proteins
JH: Juvenile hormone
PGs: Prothoracic glands
PRM: Parallel reaction monitoring
LC-MS/MS: Liquid chromatography-mass spectrometry
5F: Fifth instar feeding stage
5M: Fifth instar molting stage
6F: Sixth instar feeding stage
6M: Sixth instar metamorphic molting stage
GRIM-19: Genes associated with retinoid-IFN-induced mortality-19
IDE: Insulin-degrading enzyme
Sorcin: Soluble resistance related calcium binding protein
OBP: Odorant-binding protein-2 precursor
TRAP1: Tumor necrosis factor receptor associated protein-1
BJHSP: Basic juvenile hormone-suppressible protein 1
